# Diagnostic Value of Breast Proton Magnetic Resonance Spectroscopy at 1.5T in Different Histopathological Types

**DOI:** 10.1100/2012/508295

**Published:** 2012-05-01

**Authors:** Hyeon-Man Baek

**Affiliations:** Advanced Imaging Research Center, University of Texas Southwestern Medical Center, 5325 Harry Hines Boulevard, Dallas, TX 75390-8830, USA

## Abstract

The purpose of this study was to investigate the usefulness of quantitative proton magnetic resonance spectroscopy (^1^H-MRS) for characterizing breast lesions at 1.5T, and to evaluate the diagnostic performance of *in vivo* breast ^1^H-MRS using receiver operating characteristics (ROC) analysis. 112 patients (99 malignant and 13 benign tumors) who were scanned with the MRI/MRS protocol were included in this study. Choline-containing compounds (tCho) levels were measured and compared with histological findings. The measured tCho levels in this work had range of 0.08–9.99 mmol/kg from 65 (66%) of 99 patients with malignant tumors. Of the 13 benign lesions, ^1^H-MRS detected one as false positive, with tCho level of 0.66 mmol/kg. The optimal tCho level cutoff point that yielded the highest accuracy was found to be >0.0 mmol/kg. The resulting sensitivity was 66% and specificity 92% for distinguishing benign from malignant lesions. The tCho levels were found to be higher in invasive cancer compared to ductal carcinoma in situ or benign lesions, possibly associated with more aggressive behavior or faster cell replication in invasive cancer. Quantitative *in vivo* ^1^H-MRS may provide useful information for characterizing histopatholoigical types in breast cancer.

## 1. Introduction

High-resolution anatomic magnetic resonance imaging (MRI) and dynamic contrast-enhanced MRI have evolved into a standard clinical tool for detection and diagnosis of breast lesions [1, 2]. The morphological appearance and enhancement kinetics are two essential elements [3, 4]. However, despite its high sensitivity (94%–100%), MRI also detects many incidental enhanced lesions. The low specificity (37%–97%) may cause great anxiety to patients and many unnecessary biopsies or overtreatment [5–7]. Other adjunct imaging modalities that can better characterize the enhancing lesions on MRI are greatly needed.

*In vivo*  proton MR spectroscopy (^1^H-MRS) is a noninvasive technique that has great potential to provide tumor metabolism, which may be used in tumor diagnosis and evaluating the therapeutic response of the tumor [8–11]. Recently, breast ^1^H-MRS has been shown to improve cancer diagnosis based on elevated choline-containing compounds (tCho) metabolite peak. Several studies conducted at 1.5T have shown that *in vivo* ^1^H-MRS can be used to distinguish between benign and malignant tissues based on the hypothesis that tCho is only detectable in malignancies [8–10, 12, 13]. A pooled analysis of these studies showed that this tCho detectability criterion could identify malignancies with 89% sensitivity and 87% specificity. A similar study has been performed at higher field (e.g., 4T) showing that the increased sensitivity allows detection of tCho in benign lesions and normal subjects [14]. Levels of tCho were found to be elevated in malignancies compared to benign lesions. The sensitivity and specificity of the measurements were found to be 46% and 94%, respectively. Therefore, a more general approach is to quantify the tCho peak with the assumption that tCho levels are higher in malignancies than in benign lesions or normal tissues [14–17].

In this study, we applied an internal method using water as a reference to quantify absolute tCho levels in 112 patients with breast tumors. The purpose of this study was to investigate the usefulness of quantitative single-voxel ^1^H-MRS for characterizing breast lesions at a clinical 1.5T scanner and to evaluate the diagnostic performance of breast ^1^H-MRS using ROC (receiver operating characteristics) analysis. The performance in different tumor-size groups and between invasive cancer, in situ cancer, and benign lesions were evaluated.

## 2. Patients and Methods

### 2.1. Patients

112 patients (range 31–78 years old, mean 51 years) who were scanned with the MRI/MRS protocol were included in this study. All patients had suspicious findings on physical examination, mammography, or sonography in the breast. They were referred to the study by medical or surgical oncologists. The inclusion criteria were patients who had suspicious lesions scheduled for biopsy or who already had diagnosis of malignant breast lesions with needle biopsy. Therefore, this is a selective patient group, not in a diagnostic setting. Only lesions greater than 1 cm were scanned with the MRS protocol and included in this study. Exclusion criteria were lesions smaller than 1 cm, or with presence of a breast hematoma adjacent to the suspicious lesion. This study was approved by the institutional review board and was HIPPA compliant. The informed consent was obtained from each patient prior to the study.

The tissue diagnosis was obtained from the pathological report of the excision or needle biopsy. Of the 112 breast lesions evaluated, 99 (88%) were malignant and 13 (12%) were benign. Of the 99 malignant lesions, 66 were invasive ductal carcinoma (IDC), 10 were invasive lobular carcinoma (ILC), 12 were invasive ductal and lobular carcinoma (Mixed), and the other 11 were in situ ducal carcinoma (DCIS). Of the 13 benign lesions, 7 were fibroadenomas, 5 were fibrocystic changes, and 1 was atypical hyperplasia.

### 2.2. MR Imaging

All patients were examined with the same MRI/MRS protocol, which consisted of high-resolution imaging, dynamic contrast-enhanced MR imaging, and MR spectroscopy. The studies were performed on a clinical 1.5T whole-body system (Eclipse; Philips Medical System, Cleveland, Ohio) with the standard MRS acquisition software provided by the manufacturer. A body coil was used for transmission, and a dedicated four-channel phased-array breast coil (USA Instruments, Aurora, Ohio, USA) was used for both MR imaging and MR spectroscopy. The coil was the original coil that came with the scanner, designed to permit simultaneous imaging of both breasts. It did not allow for selective configuration of channels depending on the volume of interest.

All patients were examined in prone position, and the breasts were gently cushioned with rubber foam to reduce patient motion. After the localizer scan, sagittal view T_1_-weighted precontrast images were acquired from the breast of concern, using a spin-echo (SE) sequence with TR/TE 1000/12 ms, matrix size 256 × 256, field of view (FOV) 22 cm, and 34 slices with 3-4 mm thickness. Following this, a 3D SPGR (RF-FAST) pulse sequence with 16 frames (repetitions) was prescribed for bilateral dynamic imaging. Thirty-two axial slices with 4 mm thickness were used to cover both breasts. The imaging parameters were TR/TE 10 ms/3.6 ms, flip angle 20°, acquisition matrix size 256 × 128, and FOV varying between 32 and 38 cm. The scan time was 42 seconds per acquisition. The sequence was repeated 16 times for dynamic acquisitions, 4 precontrast, and 12 postcontrast sets. The contrast agent (Omniscan, 1 cc/10 lbs body weight) was manually injected at the beginning of the 5th acquisition and was timed to finish in 12 seconds to make the bolus length consistent for all patients. Immediately following the contrast, 10 cc saline was used to flush the contrast medium. The subtraction images were generated on the scanner console, by subtracting the pre-contrast images acquired in frame no. 3 from the 1 min postcontrast-enhanced images acquired in frame no. 6.

### 2.3. MR Spectroscopy

The subtraction images were used for placing the volume of interest for the subsequent MRS. The spectroscopic voxel was carefully positioned to maximize coverage of the enhanced lesion on the subtraction images, as well as the hypointense lesion if it was visible on the sagittal precontrast T_1_-weighted images. Localized single-voxel ^1^H-MR spectra were acquired from the enhanced lesion. The voxel size was 4.8–8.0 mL. Localization was obtained using the point-resolved spin-echo sequence (PRESS) [18, 19] followed by voxel shimming. The typical water peak linewidth (FWHM) ranged from 8 to 17 Hz. The spectra were acquired with water suppression and fat attenuation *via* three CHESS pulses [20, 21] with 60 Hz bandwidth and frequency-selective presaturation pulse (FATSAT), respectively. The following acquisition parameters were used: TR = 2000 ms, TE = 270 ms, 128 acquisitions, spectral width = 1953 Hz, and 2048 data points. A fully relaxed, unsuppressed spectrum (TR/TE = 2000/270 ms, 24 acquisitions) was also acquired to measure the amplitude of the water peak in the localized volume as the internal reference. After including the additional time for voxel placement and shimming, the total scan time for the entire sequence can be completed within 15 minutes.

### 2.4. MR Imaging Data Analysis

Measurement of tumor size was done based on the maximum intensity projection (MIP) of subtraction images. The longest dimension and the longest perpendicular dimension of the MIP were measured. The equivalent 1-dimensional tumor size was calculated by taking square root of their product. The malignant lesions were divided into three size groups as 1.0–1.9 cm (Group A), 2.0–2.9 cm (Group B), and 3.0 cm or above (Group C). The processing of subtraction, MIP, and size measurements were carried out using the “ImageJ” software (http://rsb.info.nih.gov/ij/).

### 2.5. MR Spectroscopy Data Analysis

The jMRUI software package [22] was used for time-domain analysis. For the unsuppressed spectra used to measure the water peak, each free induction decay signal was first zero-filled to 4096 points. After Fourier's transformation, automatic (or manual) phasing was used to correct every signal with the zero order phase of its water peak. The maximum peak of the water signal was assigned to 4.7 ppm, implicitly setting the polymethylene lipid peak at 1.32 ppm. For preprocessing and quantification of the water signal, we selected a frequency range of 4.2–5.2 ppm. In order to measure tCho peak from the suppressed spectrum, we performed a preprocessing that consisted of zero-filling of 4096 points, Gaussian apodization of 5 Hz, Fouriers transformation, and phase correction of the transformed spectrum. A narrow frequency range (e.g., 2.92–3.52 ppm) was selected for analyzing tCho peak to quantify its amplitude.

The spectrum was first inspected, and the signal-to-noise ratio (SNR) of the tCho peak with respect to the noise level was measured. When SNR > 2, the peak was fitted and the concentration calculated. AMARES (advanced method for accurate, robust and efficient spectral fitting) [23], a widely used quantitation tool for MRS data, was employed to fit the spectra. In this study, a Gaussian lineshape model was chosen for quantifying the tCho peak. Soft constraints were imposed for a faster and more accurate quantitation during spectral fitting. Linewidths for the tCho peak were allowed to vary between 1 and 10 Hz. The frequency constraint range was restricted to ±0.2 ppm (e.g., 3.12–3.32 ppm). After the zero- and first-order phases were switched off, the frequency-selective option [24] was applied, weighting the first 20 points of the time domain signal by the first quarter of a squared sine function. The Cramer-Rao lower bound (CRLB) was used as a measure of fitting accuracy [25]. Uncertainty in the estimated tCho concentration was the standard deviation (SD) of the tCho signal amplitude as estimated using the CRLB. In the water unsuppressed spectra, water peak was fit at 4.7 ppm.

Absolute quantification of tCho concentration was acquired using the water peak as an internal reference. All acquisitions were recorded at maximum receiver gain which made corrections for different receiver setting unnecessary. Hence, the absolute tCho concentration was calculated based on the following equation:

(1) $$\begin{array}{cc} \left[\text{tCho}\right] & =\frac{n_{{\text{H}}_{2}\text{O}}}{n_{\text{tCho}}\text{M}{\text{W}}_{{\text{H}}_{2}\text{O}}}\times \left(\frac{S_{\text{tCho}}}{S_{{\text{H}}_{2}\text{O}}}\times \frac{\sqrt{\text{N}{\text{S}}_{{\text{H}}_{2}\text{O}}}}{\sqrt{\text{N}{\text{S}}_{\text{tCho}}}}\right)\\ & \;\times \left(\frac{{f_{T_{1}}}_{{\text{H}}_{2}\text{O}}}{{f_{T_{1}}}_{\text{tCho}}}\times \frac{{f_{T_{2}}}_{{\text{H}}_{2}\text{O}}}{{f_{T_{2}}}_{\text{tCho}}}\right), \end{array}$$

where [tCho]  is the concentration of the tCho metabolite in units of mmol/kg, *S*_tCho_ is the signal amplitude of the tCho, and *S*_H_2_O_ is the signal amplitude of the unsuppressed water in the localized spectrum. The terms *n*_tCho_ and *n*_H_2_O_ represent the number of ^1^H nuclei in each respective molecule. The ratio of *S*_tCho_ and *S*_H_2_O_ amplitudes can be changed to molal concentration by correcting for the number of ^1^H nuclei per molecule and the molecular weight of water; MW_H_2_O_. $\sqrt{\text{N}{\text{S}}_{{\text{H}}_{2}\text{O}}}$ and $\sqrt{\text{N}{\text{S}}_{\text{tCho}}}$ are the numbers of data acquisitions for water unsuppressed and suppressed spectra. The parameters *f*_*T*_1__ and *f*_*T*_2__ are the correction factors for *T*_1_ and *T*_2_ relaxation times: *f*_*T*_1__ = 1 − exp⁡(−TR/*T*_1_) and *f*_*T*_2__ = exp⁡(−TE/*T*_2_). *T*_1_ relaxation times were 1513 ms for tCho and 746 ms for water; *T*_2_ relaxation times were 269 ms for tCho and 97 ms for water [16].

### 2.6. Statistical Analysis

Statistical analysis was performed using the Microcal software package (Microcal Origin Version 6.0 for Windows; Microcal Software Inc., Mass, USA). The histopathological diagnosis was used as the standard for evaluating diagnostic performance of ^1^H-MRS. An ROC curve was generated from the tCho concentration levels measured from all tumor lesions. The cut-off point of the tCho level was determined as the value that yielded the highest accuracy, balancing between sensitivity and specificity. An independent two-tailed, unequal variance *t*-test was employed to determine whether tCho detection rate and concentration level were different between invasive cancer, in situ cancer, and benign lesion groups. A *P* value <0.05 was considered statistically significant.

## 3. Results

### 3.1. Lesion Characteristics

Based on the morphological pattern of MRI enhancement, all lesions were categorized into one of two groups according to the American College of Radiology Breast Imaging Reporting and Data System lexicon (ACR BI-RADS): mass-type lesion and non-mass-type enhancements. In 99 patients with biopsy-confirmed breast cancer, cancerous lesions showed contrast enhancement in the DCE-MR imaging. Of the 99 patients with carcinoma, 78 (79%) presented a solitary mass or multiple differentiable masses with well-defined borders, and the other 21 (21%) showed nonmass enhancements without clearly defined borders. The mean tumor size of these solitary contrast-enhanced mass lesions measured on contrast-enhanced MR imaging was 2.5 cm (range 1.0–8.6 cm), whereas the size was 4.8 cm (range, 1.6–8.1 cm) for the nonmass lesions. The non-mass-type group had a significantly higher tumor size than the mass-type group (*P* < 0.0002, in Table 1). Of the 13 patients with benign lesions, 8 had mass lesions, with the mean tumor size of 1.6 cm (range, 1.1–2.3 cm), and 5 had nonmass lesions without clearly defined borders.

### 3.2. MR Spectroscopy Results

**Figure 1:**
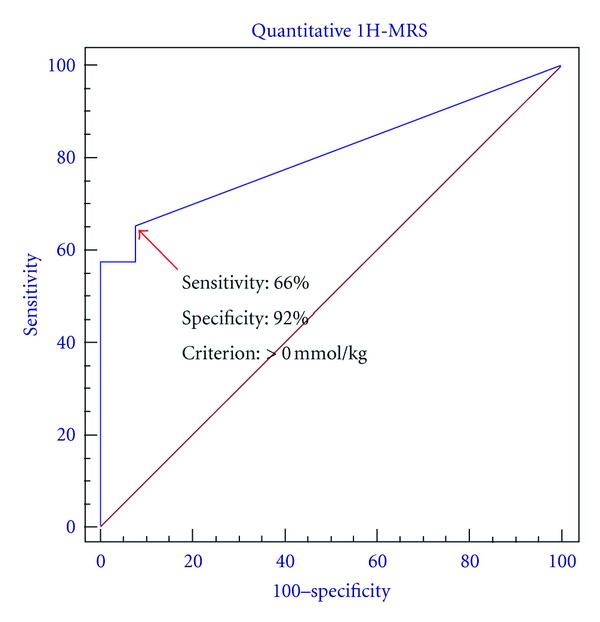
The ROC curve generated from the tCho concentration level measured in the 112 lesions (99 malignant and 13 benign). With a tCho cut-off point of >0.0 mmol/kg, the overall accuracy was 69%, with a sensitivity of 66% and a specificity of 93%.

The diagnostic performance of tCho concentration level measured by ^1^H-MRS was evaluated using the ROC analysis (Figure 1). The optimal tCho level cut-off point that yielded the highest accuracy was found to be >0.0 mmol/kg. The resulting sensitivity was 66%, specificity 92%, and overall accuracy 69% for distinguishing benign from malignant lesions. When a cutoff of 0.66 mmol/kg was selected, the sensitivity was 58%, specificity 100%, and overall accuracy 63%. The measured tCho concentration levels in this work had range of 0.08–9.99 mmol/kg from 65 (66%) of 99 patients with malignant tumors. This result was consistent with previously published value (e.g., 0.40–10.0 mmol/kg at higher field 4T) by Bolan et al. [14]. In 13 patients with benign lesions, 12 were true-negative cases (e.g., [tCho] = 0.0 mmol/kg) and the remaining one patient with fibrocystic changes was false-positive case, with tCho level of 0.66 ± 0.10 mmol/kg (Table 2).

**Figure 2:**
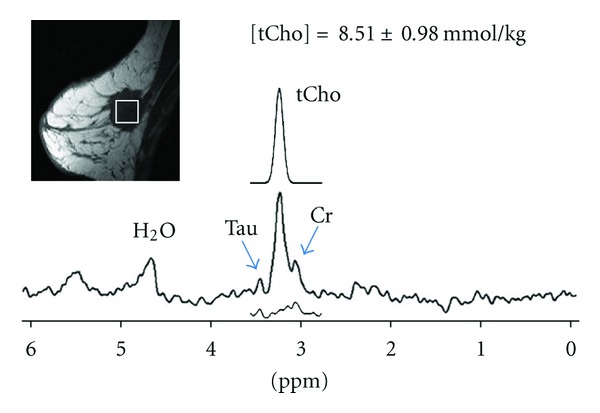
MRI and MRS measurement in a patient with mixed invasive ductal and lobular carcinoma. The equivalent 1-dimensional tumor size was 2.8 cm, and the spectroscopic voxel (size 2 × 2 × 2 cm^3^) was superimposed on the hypointense lesion in the precontrast sagittal image. The elevated tCho peak at 3.2 ppm is clearly visible in the water-fat suppressed spectrum. The Gaussian model fitting of the tCho peak produces a measurement of [tCho] = 8.51 ± 0.98 mmol/kg, the estimated model fit is shown above the full spectrum, and the residue is shown underneath. Creatine (Cr) peak at 3.02 ppm and taurine (Tau) peak at 3.45 ppm are also observed.

**Figure 3:**
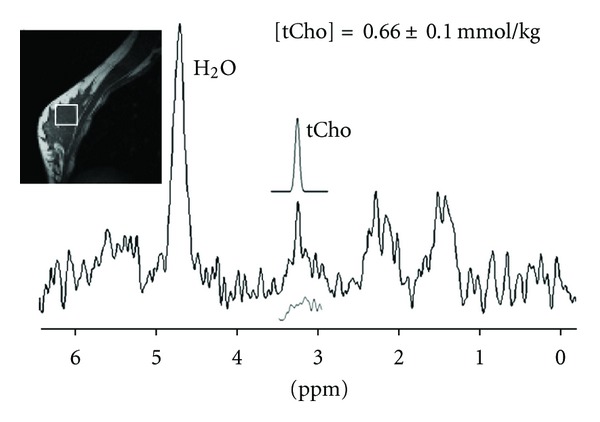
MRI and MRS measurement in a patient with false-positive benign lesion. This patient showed dense glandular tissues on the sagittal precontrast image and mild enhancement on the enhanced MR image. The spectroscopic voxel size was 2 × 2 × 2 cm^3^. The elevated tCho peak at 3.2 ppm is clearly visible in the water-fat suppressed spectrum. The Gaussian model fitting of the tCho peak yields a measurement of [tCho] = 0.66 ± 0.10 mmol/kg, the estimated model fit is shown above the full spectrum, and the residue is shown underneath.

Figure 2 shows representative MRI and ^1^H-MRS on a patient diagnosed by histology with breast cancer (e.g., mixed invasive ductal and lobular carcinoma). The lesion size was 2.8 cm, and spectroscopic voxel (size, 2 × 2 × 2 cm^3^) was carefully positioned to maximize the coverage of the hypointense lesion on the centered sagittal image and on the contrast-enhanced lesion in the subtraction axial image. The elevated tCho peak at 3.22 ppm is clearly detected in the water-fat suppressed spectrum. The Gaussian model fitting produces a measurement of [tCho] = 8.51 ± 0.98 mmol/kg, the estimated model fit is shown above the full spectrum, and the residue is shown underneath.  Figure 3 shows MRI and ^1^H-MRS from a patient with a benign fibrocystic changes. This patient showed dense glandular tissues on the sagittal-view precontrast image, and on the axial view subtraction image a heterogeneous enhancement area was noted in the posterior right breast. ^1^H-MRS demonstrated an increased tCho peak in the enhanced areas. The Gaussian model fitting of the tCho peak yields a measurement of [tCho] = 0.66 ± 0.10 mmol/kg, and the residue is shown underneath.

**Figure 4:**
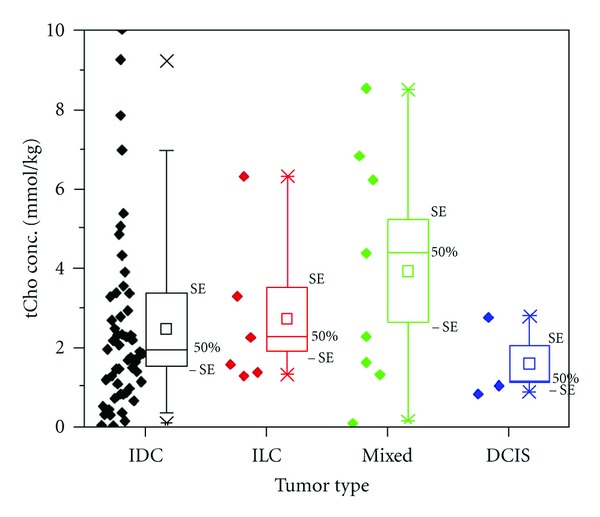
Plot of tCho concentration versus tumor type. The tCho concentration level was measured from 65 true-positive lesions, and the range was from 0.08–9.99 (mean ± SD, 2.7 ± 2.3 mmol/kg), consistent with the previously published value. The mean tCho levels were IDC = 2.49, ILC = 2.70, mixed = 3.92, and DCIS = 1.57 mmol/kg. The mean tCho levels were found to be higher in invasive cancer compared to noninvasive cancer, possibly associated with more aggressive behavior or faster cell replication in invasive cancer.

Figure 4 shows the tCho levels of all individual patients in each of the four groups. A large variation was observed in all groups. No significant group differences were observed in the tCho concentration levels (*P* > 0.05). The mean values were 2.49 for IDC, 2.70 for ILC, 3.92 for mixed, and 1.57 mmol/kg for DCIS, respectively.

**Figure 5:**
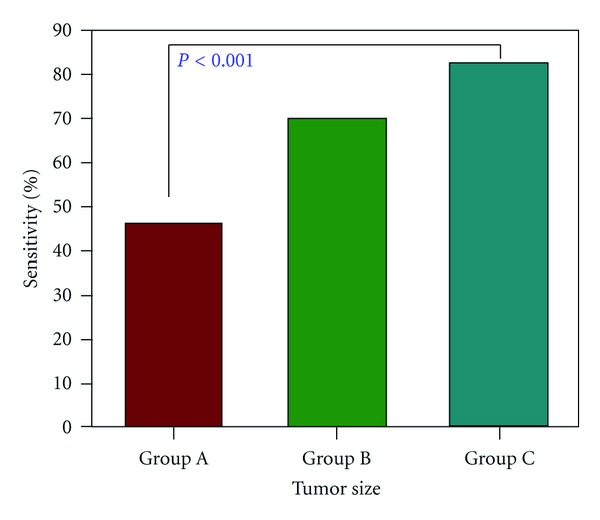
Sensitivity of breast ^1^H-MRS in different tumor size groups. The sensitivity increased from 46% (Group A, 1.0–1.9 cm), to 70% (Group B, 2.0–2.9 cm), and 82% (Group C, 3.0 cm and above), in a statistically significant manner (*P* < 0.0001, two-sided exact Kruskal-Wallis test).

**Figure 6:**
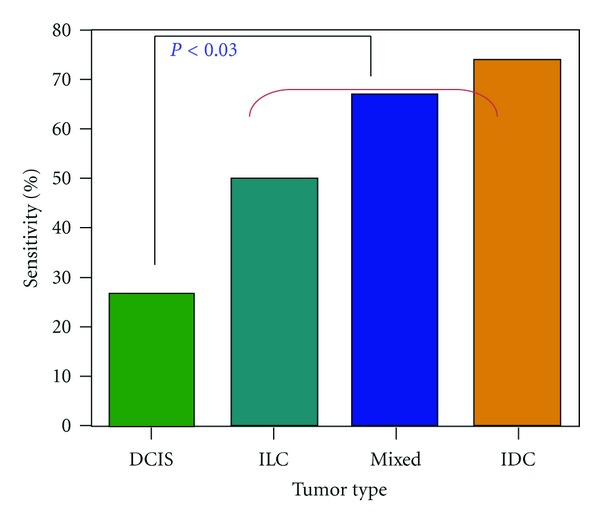
Sensitivity of breast ^1^H-MRS in different histopathological lesion groups. The sensitivity was significantly higher in invasive cancer than in noninvasive cancer, ductal carcinoma in situ (*P* < 0.03). The sensitivity was higher in IDC compared to ILC, but not significant (*P* > 0.05).

Figure 5 shows the sensitivity of breast ^1^H-MRS in malignant tumor size groups. The sensitivity increased from 46% (16/35, Group A), to 70% (21/30, Group B), to 82% (28/34, Group C), in a statistically significant manner (*P* < 0.0001, two-sided exact Kruskal-Wallis test). The overall sensitivity was 66% (65/99). When a smaller size was chosen (<1.5 cm), the sensitivity was further decreased (29%, 4/14).  Figure 6 shows the sensitivity of ^1^H-MRS in histopathological groups. The sensitivity was significantly higher in invasive cancer (71%, 62/88) than in noninvasive cancer, DCIS (27%, 3/11) (*P* < 0.03). Among invasive cancers, the ^1^H-MRS sensitivity was higher in IDC (74%, 49/66) compared to ILC (50%, 5/10), but not significant (*P* > 0.05).

## 4. Discussion

*In vivo* ^1^H-MRS is a noninvasive technique that has great potential to provide complementary information to improve breast cancer diagnosis. The diagnostic value of *in vivo*  breast MR spectroscopy is typically based on the detection of elevated level of tCho, which is a marker of active tumor. Quantitative measurement of tCho may improve the accuracy of lesion diagnosis because the sensitivity of ^1^H-MRS is variable due to variations in voxel size, adipose tissue content, and receiver coil efficiency. The present study analyzed the quantitative single-voxel ^1^H-MRS data using the ROC analysis and set the criteria based on the cut-off point as tCho level >0.0 mmol/kg. Using this criterion, the sensitivity and specificity were 66% and 92%, respectively. They were in the similar range as reported in previously published study [14].

The measured tCho concentration levels in this work were within a range of 0.08–9.99 (mean ± SD, 2.7 ± 2.3 mmol/kg) from 66 of 99 patients with malignant breast tumors using 1.5T, which are consistent with the findings of previous published results. Roebuck et al. [8] found tCho levels ranging 0.4–5.8 mmol/L in seven patients with confirmed malignant breast tumors. Bakken et al. [15] reported 2.0 mM of choline-containing compounds found in a single breast cancer patient. Baek et al. [17] reported that the tCho levels had a range of 0.32–10.47 mmol/kg from 32 patient with malignant breast tumors at 1.5T. Bolan et al. [14] reported a range of tCho measurements of 0.40–10.0 mmol/kg in 71 malignant breast tissue spectra at 4T. The large range in tCho concentration levels for malignant lesions was observed. This result may reflect the heterogeneous nature of breast lesions. Gribbestad et al. [26] reported that phosphatidylcholine, a precursor of choline-derived phospholipids, also showed a large variation even among the same tumor types. Ting et al. [27] reported that the increase of choline has often been reported in breast cancer and is regarded as a marker for elevated proliferation rates. Singer et al. [28] reported that the metastatic breast cancer cell line 21MT-2 had a significantly higher concentration of choline than did the primary breast cancer cell lines 21PT and 21NT.

In this study, ^1^H-MRS detected one of 13 patients with benign lesion as a false-positive case, with tCho level of 0.66 mmol/kg (Figure 3). In some of the previous studies, tCho peaks were detected in benign lesions at 1.5T: Roebuck et al. [8] (one case), Kvistad et al. [9] (two cases), Jagannathan et al. [10] (eight cases), Yeung et al. [12] (one case), and Cecil et al. [13] (two cases). Stanwell and Mountford [29] also reported detecting tCho peak in false-positive volunteers or lactating mothers at 1.5T. Thus, these false-positive cases probably represent the actual limits of the specificity of breast ^1^H-MRS in diagnosis of breast cancer. Recently, Haddadin et al. [30] reported that tCho concentration level was higher in cancers than in benign lesions or normal breast tissues at 4T high field. Comparing only the benign and malignant measurements, an ROC analysis led to a cutoff of 1.45 mmol/kg, giving 73% sensitivity and 77% specificity for distinguishing benign from malignant lesions. For *in vivo* ^1^H-MRS using 1.5T, however, further investigation in a large population (e.g., benign lesions) is needed to determine a threshold tCho value for differentiating between malignant and benign lesions.

*In vivo* ^1^H-MRS studies have demonstrated that elevated tCho peak at 3.2 ppm is observed in neoplastic tissues [8, 9, 12, 13]. However, the precise mechanism for elevated tCho is not completely understood and still remains unclear. High-resolution ^1^H-NMR spectra acquired from biopsy tissues have shown that a tCho resonance peak actually is comprised of multiple signals, such as phosphocholine, glycerophosphocholine, and free choline [31]. These three signals cannot be resolved *in vivo*  at 1.5T, at which only a single resonance peak, representing the aggregate of all choline-containing compounds, is observed. Among these signals, the primary component contributing to the tCho peak is phosphocholine, a known precursor of cell membranes synthesis [26, 27, 32]. The increased choline kinase (ChoK) and its product, phosphocholine, have been implicated in human carcinogenesis [33]. Thus, the elevated tCho level in breast cancer may be associated with increased membrane synthesis by replicating cells. However, benign tissues such as proliferative fibroadenomas may also show a positive tCho signal [12].

In this study, tCho detection rate was higher in invasive cancer (e.g., IDC, ILC, and mixed) compared to DCIS, possibly associated with more aggressive behavior or faster cell replication (Figure 4). This finding is consistent with previously reported results, showing that, on *in vivo* ^1^H-MRS, IDC was consistently positive for tCho detection, whereas DCIS was likely negative [34]. 3 (27%) of 11 patients with DCIS in our study were positive for tCho detection, but the remaining 8 (73%) patients had false-negative cases. The tCho detection rate was also higher in IDC (75%, 49/65) compared to ILC (55%, 6/11), which was possibly related to the infiltrating phenotype of ILC, thus, more susceptible to the fat contamination problem (Table 2). However, there was no significant difference in the tCho detection rate and concentration level between invasive cancers, which is consistent with the findings of previous study [35]. The lipid contamination in ^1^H-MRS voxel may attribute to low sensitivity [17]. The adipose tissue limits the ability to optimize the field homogeneity inside the selected ^1^H-MRS volume, which in turn leads to broad resonance peaks and reduced SNR. Recently, Thakur et al. [35] reported that water-to-fat (W/F) ratio measure from *in vivo* ^1^H-MRS may be useful to differentiate IDC from ILC tumors because the ratio was significantly higher in the IDC lesions compared to the ILC lesions (*P* < 0.001). IDC tumors form focal masses and obliterate background tissue as it grows, while ILC tumors are connected as a single strand, and these invade tissue without formation of focal mass, preserving background fat [36, 37].

Of the 99 malignant lesions, there were 34 false-negative cases, which resulted in 66% sensitivity (Table 2). This might be due to partial volume effects from intermixed tumor and normal tissues in a ^1^H-MRS voxel. Due to this problem, *in vivo*  single-voxel ^1^H-MRS will have a very limited role for characterizing small lesions. When the data were divided into three size groups (e.g., <2.0, 2.0–2.9, ≥3.0 cm), the sensitivity of the ^1^H-MRS in these size-dependent subgroups increased from 46% to 70% and to 82%, in a statistically significant manner (Figure 5). More than half of lesions smaller than 2.0 cm (54%) had false-negative diagnosis, because of the lack of a detectable tCho signal. When a smaller size was chosen (<1.5 cm), the sensitivity was further decreased (29%, 4/14). Therefore, further improvement in the signal-to-noise ratio may enhance the detection of tCho and improve the diagnostic sensitivity. One approach is to use the scanner at a higher magnetic field, but it may suffer from a worse field inhomogeneity problem. No large cohort study has been reported from 3T yet.

In conclusion, in this study we reported diagnostic value of quantitative *in vivo* ^1^H-MRS spectroscopy at 1.5T in different histopathological types. On the basis of that threshold (e.g., >0.66 mmol/kg), ^1^H-MRS alone presented 58% sensitivity and 100% specificity for diagnosis. The tCho levels were found to be higher in invasive cancer compared to DCIS or benign lesions, possibly associated with more aggressive behavior or faster cell replication in invasive cancer. Therefore, quantitative ^1^H-MRS may provide useful information for characterizing tumor types of breast cancer. However, *in vivo* ^1^H-MRS at 1.5T has a limited role for characterizing small lesions.

## Figures and Tables

**Table 1:** Summary of tumor size, tCho detection rate, and tCho concentration level in morphological types.

Tumor morphology	Mean size (cm)**	No. of true positives	No. of false negatives	tCho detection rate	Mean tCho (mmol/kg)
Mass	2.5	53	25	82%	2.76
Nonmass	4.8	12	9	57%	2.39

Tumor size (cm) was calculated by taking the square root of the product (e.g., the longest dimension × the longest perpendicular dimension of the maximum intensity projection (MIP) of the MR subtraction images). **P < 0.0002, where the significance level was set at *P* < 0.05. There is no significant group difference in tCho level (*P* > 0.05).

**Table 2:** Summary of tumor size, tCho detection rate, and tCho concentration level in histopathological types.

Tumor type	Mean size (cm)	No. of true positives	No. of false negatives	tCho detection rate*	Mean tCho (mmol/kg)
IC	2.9	62	26	71%	2.65

IDC	3.2	49	17	74%	2.49
ILC	2.3	5	5	50%	2.70
Mixed	2.4	8	4	67%	3.92

DCIS	2.4	3	8	27%	1.57
Benign	1.6	1 (false+)	12 (true−)		0.66

IC: invasive cancer, IDC: invasive ductal carcinoma, ILC: invasive lobular carcinoma, mixed: mixed invasive ductal and lobular carcinoma, and DCIS: ductal carcinoma in situ. *P = 0.025 (IC versus DCIS), where the significance level was set at *P* < 0.05.
